# A sustained release of alendronate from an injectable tetra-PEG hydrogel for efficient bone repair

**DOI:** 10.3389/fbioe.2022.961227

**Published:** 2022-09-13

**Authors:** Shuai Chang, Chao Li, Nanfang Xu, Jiedong Wang, Zehao Jing, Hong Cai, Yun Tian, Shaobo Wang, Zhongjun Liu, Xing Wang

**Affiliations:** ^1^ Department of Orthopedics, Peking University Third Hospital, Beijing, China; ^2^ Beijing Key Laboratory of Spinal Disease Research, Peking University Third Hospital, Beijing, China; ^3^ Engineering Research Center of Bone and Joint Precision Medicine, Ministry of Education, Peking University Third Hospital, Beijing, China; ^4^ Beijing National Laboratory for Molecular Sciences, Institute of Chemistry, Chinese Academy of Sciences, Beijing, China; ^5^ Senior Department of Orthopedics, the Fourth Medical Center of PLA General Hospital, Beijing, China; ^6^ University of Chinese Academy of Sciences, Beijing, China

**Keywords:** tetra-PEG hydrogel, AlN, bone regeneration, drug delivery, injection

## Abstract

Significant efforts on construction of smart drug delivery for developing minimally invasive gelling system to prolong local delivery of bisphosphonates are considered as promising perspectives for the bone-related diseases, which provide the hydrogels with unique bioactivities for bone repair in clinic. Herein, we have constructed an alendronate (ALN)-conjoined injectable tetra-PEG hydrogel with excellent biocompatibility, uniform network, and favorable mechanical properties in one-pot strategy. In views of the quick ammonolysis reaction between N-hydroxysuccinimide (NHS)-ester of tetra-PEG-SG and amine groups of tetra-PEG-NH_2_ polymer and ALN molecules, the uniform networks were formed within seconds along with the easy injection, favorable biocompatibility and mechanical properties for hydrogel scaffolds. On account of the simultaneous physical encapsulation and chemical linkage of the ALN within the hydrogels, the ALN-conjoined tetra-PEG hydrogel exhibited a sustained drug release delivery that could persistently and effectively facilitate viability, growth, proliferation, and osteogenesis differentiation of stem cells, thereby allowing the consequent adaptation of hydrogels into the bone defects with irregular shapes, which endowed the ALN-conjoined tetra-PEG hydrogel with depot formulation capacity for governing the on-demand release of ALN drugs. Consequently, the findings imply that these drug-based tetra-PEG hydrogels mediate optimal release of therapeutic cargoes and effective promotion of *in situ* bone regeneration, which will be broadly utilized as therapeutic scaffolds in tissue engineering and regenerative medicine.

## Introduction

Bone defects from trauma and tumor resection are a kind of clinical diseases that can cause many severe problems to reduce the life quality (Service, 2000; Senarath-Yapa et al., 2016; Vi et al., 2018; Zhang et al., 2022). Although surgical operations like autologous and allogeneic bone grafting are the most clinically used strategies for bone repair, the donor shortage, immune rejection and insufficient transplantation materials always greatly limit their application (Bauer and Muschler, 2000; Dimitriou et al., 2011; Wang and Yeung, 2017). To tackle these shortcomings, engineering technology has opened new avenues for promoting bone repair and tissue regeneration based on the many advantages of exogenous progenitor cells and controlled release of bioactive factors (Langer and Vacanti, 1993; Petite et al., 2000; Wang et al., 2019; Cheng et al., 2021a; Fu et al., 2022). Therefore, bone tissue engineering is developed as an alternative to enhance the proliferation and differentiation of osteoblast using the osteoinductive scaffolds. Despite rapid progress over the past 20 years, the mechanical properties of engineered scaffolds are generally not unqualified as natural tissues and cannot be used as bone graft materials.

Hydrogels are comprised of cross-linked polymeric networks with designable chemical architectures and uniquely physical properties, which can be divided into naturally derived hydrogels and synthetic hydrogels (Kim et al., 2017; Wang et al., 2020; Yu et al., 2020; Cheng et al., 2021b; Yang et al., 2022). Until now, naturally derived hydrogels with excellent biocompatibility, good biodegradability, easy accessibility and renewable sources have been widely explored as the bone regenerative scaffolds to emulate extracellular matrices and improve the cell viability, proliferation, and differentiation for bone regeneration. However, their inhomogeneous networks always lead to the poor mechanical properties and machinability that can greatly hinder their therapy of bone defect (Zhai et al., 2019; Tang et al., 2020a; Tang et al., 2020b; Zou et al., 2021). In compassion, synthetic hydrogels possess designable architectures and tunable properties (degradation time, mechanics, machinability, responsibility, etc.) for constructing a varied of multifunctional and high-performance scaffolds, thus enabling the advantage of simple drug formulation and the ability to adjust the hydrophilic drugs delivery (Yang et al., 2018; Xu et al., 2023). With the aim of repairing bone tissues with the assistance of synthetic hydrogels, much progress has been made in furnishing them with robust mechanical properties in new approaches. As an outstanding representative, tetra-PEG hydrogel is recognized as an ideal homogeneous biomaterial due to the excellent biocompatibility, essential non-immunogenicity, and facilely chemical modification for construction of multifunctional hydrogel scaffolds in a convenient way for the widely clinic applications (Sakai et al., 2008; Konieczynska et al., 2016; Li et al., 2020; Zhu et al., 2022).

Mesenchymal stem cells are existing in multiple tissue types, including the craniofacial and dental tissues. It has been reported that orofacial tissues derived MSCs obtained superior proliferation, immunomodulation, and multiple-lineage differentiation abilities when compared with bone marrow derived mesenchymal stem cells (BMMSCs). Periodontal ligament stem cells (PDLSCs) isolated from the periodontal ligament can differentiate into many types of specialized cells, including osteoblast-like cells that contribute to periodontal tissue repair. Compared with other types of derived MSCs, ex vivo-expanded PDLSCs could be easily collected in clinic from discarded tissue samples and achieve good bone regeneration capacity. In addition, several recent studies have focused on the relationship of estrogen deficiency-induced osteoporosis with periodontal diseases, and demonstrated estrogen can promote the osteogenic differentiation of PDLSCs in osteoporotic rats (Li et al., 2014; Xu et al., 2016), which help in the development of a potential therapeutic strategy for periodontal disease in postmenopausal women.

**FIGURE 1:**
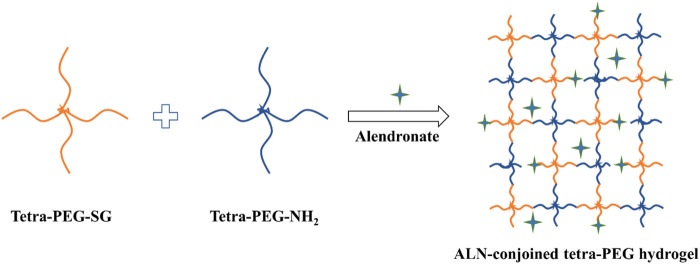
Schematic illustration of the fabricated ALN-conjoined tetra-PEG hydrogel.

As famous well-known drugs, bisphosphonate (BPs) can prevent bone loss in patients because of their remarkable selectivity to bone rather than other tissues, which are developed as a good choice for the therapy of various bone-related disease, e. g., post-menopausal osteoporosis, Paget’s disease, metastasis to bone and hypercalcemia. In addition, since BP groups can strongly and reversibly bind calcium ions and can also bind to hydroxyapatite, calcium phosphate and other calcium crystals, bisphosphonates displayed a high affinity for bone-related minerals, which were reported to upregulate alkaline phosphatase and mineralization and promote the expression levels of osteogenic-related genes in osteoblasts and improve osteogenic differentiation of stem cells to facilitating the bone defects repair (Cramer et al., 2007; Wang et al., 2015; Shi et al., 2017; Liu et al., 2018; Shi et al., 2019; Kuznik et al., 2020). As a typical representative of bone resorption inhibitor, alendronate (ALN) is a most prescribed oral amino-bisphosphonate drug through inhibiting the resorptive activity for the bone therapy. However, its high hydrophilicity leads to the poor oral bioavailability and gastrointestinal permeability (Zhang et al., 2017; Wang et al., 2021). Consequently, developing intelligent drug formulation to promote osteogenesis has been a promising candidate for clinical treatment of osteoporosis and other bone-related diseases. In the present study, we designed and constructed an injectable ALN-conjoined tetra-PEG hydrogel via the mixing 4-armed poly (ethylene glycol) succinimide glutarate ester (tetra-PEG-SG), 4-arm poly (ethylene glycol) amine (tetra-PEG-NH_2_) and ALN drug into aqueous solutions *in situ* for facilitating osteogenesis and bone repair (Figure 1). The amine groups of tetra-PEG-NH_2_ polymer could rapidly react with N-hydroxysuccinimide (NHS)-ester of tetra-PEG-SG polymer to form the crosslinking amide bonds and uniform networks within seconds, contributing to the highly mechanical properties. Meanwhile, it was possible that the amine groups of ALN drugs could also steadily react with tetra-PEG-SG to bind onto the hydrogels, thus leading to the favorable osteogenesis capacities. In views of this simultaneous physical encapsulation and chemical linkage of the ALN within the hydrogels, this smart system can exhibit a sustained release delivery and elucidate the therapeutic efficiency on facilitating cell viability, growth and proliferation as well as the osteogenic differentiation and bone regeneration.

## Materials and methods

### Materials

4-arm poly (ethylene glycol) (tetra-PEG-OH, *M*_w_ = 10 kDa, *M*_w_/*M*_n_ = 1.03) and 4-arm PEG-amine (tetra-PEG-NH_2_, *M*_w_ = 10 kDa, *M*_w_/*M*_n_ = 1.03) were purchased from SINOPEG, China. Glutaric anhydride (98%), N-hydroxysuccinimide (NHS, 98%) and alendronate (ALN, 99%) were purchased from Energy Chemical. All other reagents were purchased from Beijing Chemical Works and used as received without further purification. PDLSCs were supplied by China Infrastructure of Cell Line Resource.

### Measurements

^1^H NMR spectra were obtained on a Bruker DRX-400 spectrometer using tetramethylsilane (TMS) as internal reference. Scanning electron microscopy (SEM) images were obtained at acceleration voltage of 5 kV on a JSM-6700F microscope (JEOL, Japan). These samples were directly sputter-coated with a thin layer of Pt for 120 s to make the samples conductive before the testing. Rheological behaviors were conducted on a rheometer (Thermo Haake Rheometer, Newington, NH, United States). During the experiments, the ALN-conjoined tetra-PEG hydrogels were spread on a parallel plate (25 mm) and sealed with silicone oil to prevent solvent evaporation. The compressive profiles of hydrogels were measured using a testing machine of Instron 3,365 (Instron Co., Norwood, MA, United States), and the hydrogel samples were cut into cylinders for the compressive testing with a beam velocity of 1 mm/min. Confocal laser scanning microscopy (CLSM) images were obtained on a Zeiss LSM 510 microscope. The ALN concentration was measured by high performance liquid chromatography (HPLC) on a Shimadzu LC-20AT system with UV detection at 266 nm. A mixture of acetonitrile and water (v/v = 1/3) was used as mobile phase at a flow rate of 1.0 ml/min.

### Synthesis of tetra-PEG-SG polymer

First, the intermediate product tetra-arm PEG-glutaric acid (tetra-PEG-COOH) was prepared by reacting tetra-PEG-OH (1 g, 0.1 mmol), glutaric anhydride (114 mg, 1 mmol) and DMAP (122 mg, 1 mmol) in 25 ml of anhydrous CH_2_Cl_2_ for 12 h. After that, the solution was washed with 2 M HCl aqueous solution, saturated NaCl aqueous solution and DI water for three times, and dried over anhydrous MgSO_4_. The final product was further precipitated into diethyl ether for several times to afford the white powder of tetra-PEG-COOH polymer under vacuum.

Then tetra-PEG-COOH (1 g, 0.1 mmol), EDCI (384 mg, 2 mmol) and NHS (230 mg, 2 mmol) were dissolved in 25 ml of anhydrous CH_2_Cl_2_ for 12 h. After that, the solution was washed with 2 M HCl aqueous solution, saturated NaCl aqueous solution and DI water for three times, and dried over anhydrous MgSO_4_ to afford the white solid of tetra-PEG-SG polymer under vacuum.

### Synthesis of mPEG-ALN

mPEG-COOH (0.85 g, 1 mmol), EDCI (384 mg, 2 mmol) and NHS (230 mg, 2 mmol) were dissolved in 25 ml of anhydrous CH_2_Cl_2_ for 12 h. After that, the solution was washed with 2 M HCl aqueous solution, saturated NaCl aqueous solution and DI water for three times, and dried over anhydrous MgSO_4_ to afford the white solid of mPEG-NHS polymer under vacuum.

Then ALN (0.33 g, 1 mmol) and mPEG-NHS (1.22 g, 1 mmol) was added to a 25 ml round-bottomed flask equipped with a magnetic stirring bar followed by the addition of 10 ml of Na_2_CO_3_ solution to fully dissolve all the solids. After the reaction for 18 h, the unreacted monomers and other impurities were removed by the dialysis (MW cutoff, 500 Da) against deionized water for 2 days and collected after freeze-drying to afford the white powder of mPEG-ALN under vacuum.

### Preparation of tetra-PEG hydrogel

Tetra-PEG-NH_2_ (120 mg) polymer was dissolved in a 1 ml of bottle to form a precursor solution, and tetra-PEG-SG (120 mg) polymer was dissolved in another 1 ml of bottle to form another precursor solution. By using dual syringe, same volumes of precursor solutions were simultaneously injected into the moulds at room temperature to quickly form the tetra-PEG hydrogel with the gelation time of less than 10 s.

### Preparation of ALN-conjoined tetra-PEG hydrogel

Tetra-PEG-NH_2_ (120 mg) polymer and ALN (3 mg) were dissolved in a 1 ml of bottle to form a precursor solution, and tetra-PEG-SG (120 mg) polymer was dissolved in another 1 ml of bottle to form another precursor solution. By using dual syringe, same volumes of precursor solutions were simultaneously injected into the moulds at room temperature to form the ALN-conjoined tetra-PEG hydrogel with the gelation time of less than 10 s.

### *In vitro* drug release from the hydrogel

The hydrogel sample was prepared in a container with the diameter of 10 mm and height of 2 mm, and the ALN-conjoined tetra-PEG hydrogel was immersed into the PBS (pH 7.4) and the solutions were collected at special intervals of time to test the ALN concentration using the HPLC.

### *In vitro* cytotoxicity assay

The cell viability was studied by CCK-8 cytotoxicity assay. The PDLSCs were suspended in cell culture medium and seeded into 48-well plates with a density of 1 × 10^4^ cells/100 µL in each well and incubated for 24 h at 37°C in a 5% CO_2_ humidified incubator. The hydrogel samples were immersed in the fresh cell medium (10 ml) for 24 h to get the extracts, and then the treated cell medium was used to replace the fresh cell medium and the cells were further incubated for an additional time. Cells cultured in fresh medium were used as control. Cell number was correlated with optical density (OD). Cell viability (%) was calculated using the following Equation:

$$\mathrm{Cell}\;\mathrm{viability}\left(\%\right)=\left[\left(A_{\mathrm{sample}}-A_{\mathrm{blank}}\right)/\left(A_{\mathrm{control}}-A_{\mathrm{blank}}\right)\right]\times 100\%$$ (1)

### Live/dead staining assay

Cell live and dead viability was determined by Live/dead viability according to the manufacturer’s instruction. The staining reagent mixture, a red fluorescent propidium iodide (PI) stain and a green fluorescent (AM) stain were added to the mixture and incubated for 15 min. The corresponding fluorescence emission of PDLSCs was then assessed using confocal laser scanning microscopy (CLSM).

### Cell proliferation assay

Cell proliferation was measured using CCK-8. PDLSCs were seeded and incubated in growth medium for 24 h, hydrogel sample was added and incubated for another 24 h. After 1–5 days of incubation, the cell culture medium was removed and then 100 µL of fresh culture medium and 10 µL of CCK-8 were added to the 96 wells for 4 h. Finally, the absorbance was read at 450 nm on a microplate reader (Thermo, Waltham, MA, United States). Cell number was correlated with OD value, and the OD value could calculate the cell proliferation.

### Osteogenesis-related gene expression

Quantitative reverse transcription polymerase chain reaction (RT-qPCR) was performed in a RT-qPCR device to analyze the relative expression of osteogenesis-related genes including ALP, Runt-related transcription factor 2 (RUNX2), collagen type 1 (COL 1) and osteocalcin (OCN). All the cycle threshold values of the genes were normalized to their internal reference and quantitatively analyzed.

### Statistics analysis

All results were presented as mean and standard deviation with 3-6 independent experiments. The statistics were analyzed using the SPSS software. When *p* < 0.05, differences were considered to be significant (*p < 0.05, **p < 0.01).

## Results and discussion

### Preparation and characterization of tetra-PEG and ALN-conjoined tetra-PEG hydrogels

**FIGURE 2:**
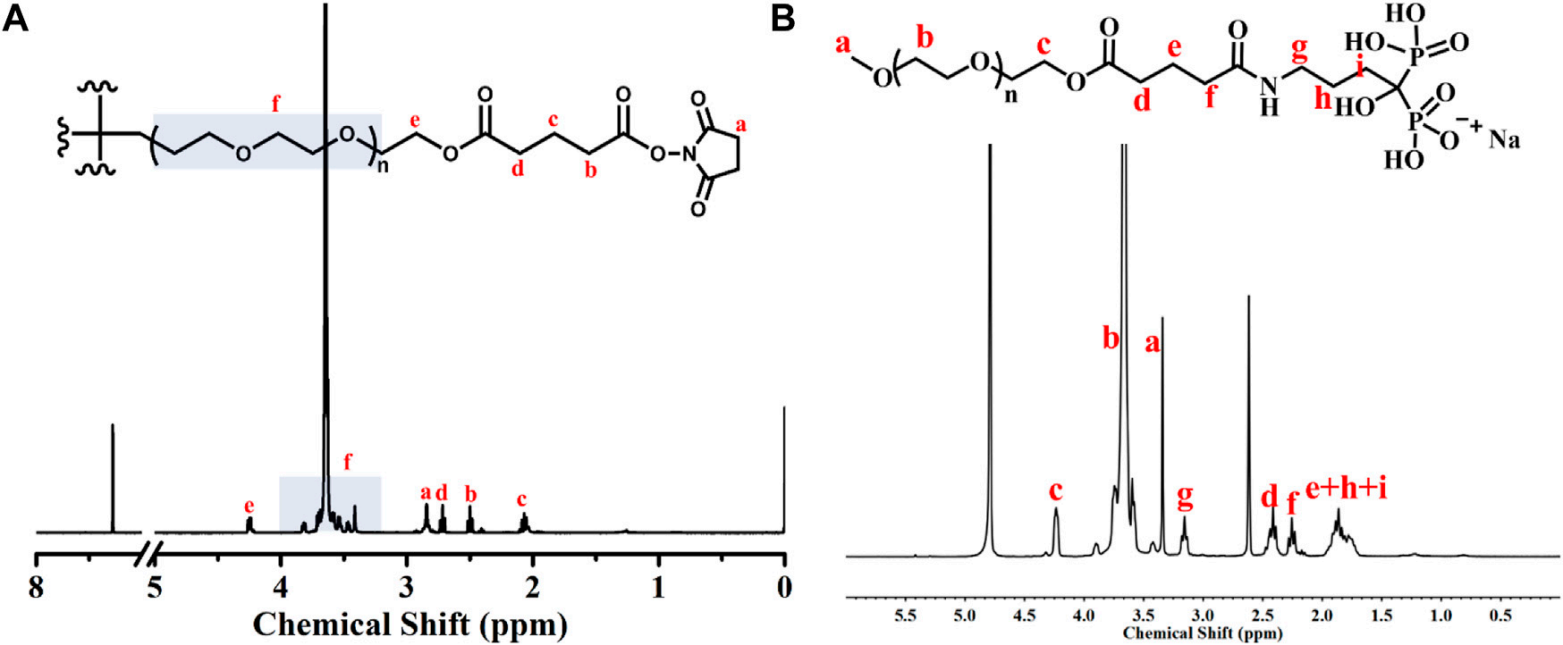
^1^H NMR spectra of **(A)** tetra-PEG-SG and **(B)** mPEG-ALN polymers.

The schematic illustration of ALN-conjoined tetra-PEG hydrogel was clearly demonstrated in Figure 1. Tetra-PEG-SG polymer was easily synthesized in two steps without the misgivings of being inhibited by anticoagulation agents and transferring disease. All the peaks in the ^1^H NMR spectrum (Figure 2A) can be justly attributed to the explicit structure and the integration ratio of peak a, b, c and d nearly equals to 2:1:1:1, demonstrating the successful synthesis of tetra-PEG-SG polymer. In views of highly effective ammonolysis reaction between the amine and active ester groups, an injectable tetra-PEG hydrogel was quickly formed within 10 s through mixing the tetra-PEG-NH_2_ and tetra-PEG-SG solutions using a dual syringe. Before the gelation, the simultaneous addition of ALN drug can be both physically encapsulated and chemically linked into the tetra-PEG hydrogel because of the terminated amine groups, which was defined as ALN-conjoined tetra-PEG hydrogel.

To prove the effective ammonolysis reaction between the tetra-PEG-SG and ALN, we prepared a liner polymer of mPEG-ALN by mixing these components in a short time, and structural attribution in Figure 2B revealed the quick formation of chemical linkage in solutions as well as the chemical anchor onto the tetra-PEG hydrogels. It is mentioned that during this gelling process, high adhesion strength onto the tissue could be meanwhile achieved via the formation of chemical linkage among the amine groups of proteins within the tissue and tetra-PEG-SG polymer, demonstrating its facile application in the bone-related disease with advantages of easy injection, quick gelation and high tissue adhesion. In addition, according to the previous literatures, alendronate-modified hydrogels could markedly accelerate the osteoblastic differentiation and increase osteogenesis in both 2D and 3D environments. More importantly, low-dose of alendronate (<1.5 mmol) can serve as an optimal osteo-inductive factor to promote osteogenesis *in vitro* and accelerate the osteoblastic differentiation of cells without the need for osteogenesis inducing supplements for bone regeneration *in vivo* (Wang et al., 2009; Wang et al., 2010; Liu et al., 2018). In addition, on account of the quick reaction between amine groups of ALN drugs and NHS-ester groups of tetra-PEG-SG polymer, the loading rate can be nearly precise calculated within the ALN-conjoined tetra-PEG hydrogel. Therefore, we have directly used the low drug concentration of ca. 1 mmol and not further focused on exploring the effect of ALN concentration on the osteogenesis.

**FIGURE 3:**
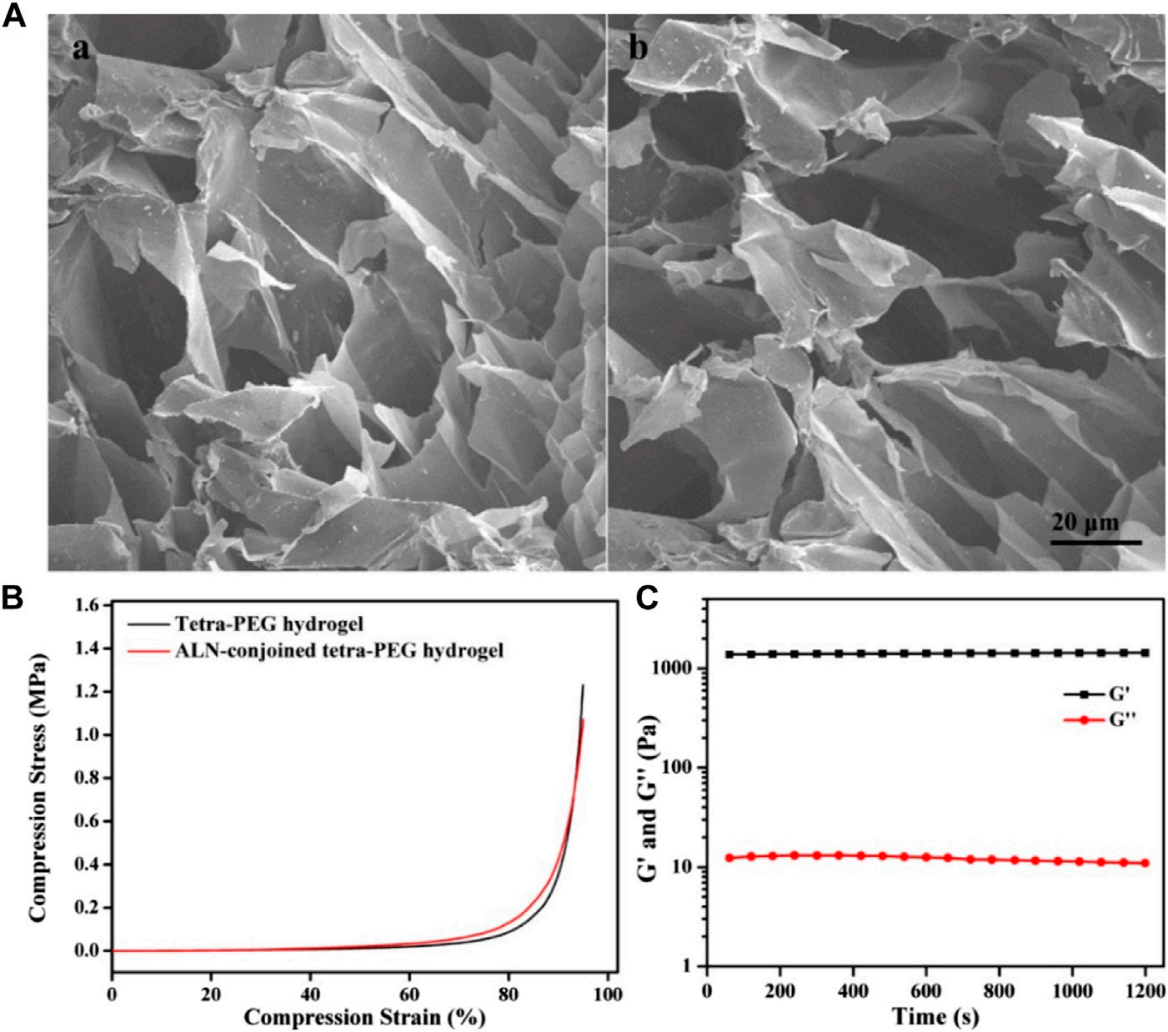
**(A)** SEM images of **(A)** tetra-PEG and **(B)** ALN-conjoined tetra-PEG hydrogels. **(B)** Compressive curves of tetra-PEG hydrogels with or without ALN loading. **(C)** Rheological profile of ALN-conjoined tetra-PEG hydrogel.

The morphologies of hydrogels were observed by SEM images in Figure 3A, which showed the similar inner pores of tetra-PEG and ALN-conjoined tetra-PEG hydrogels without significant difference. These hydrogel scaffolds possessed large pore size and homogeneous porosity that could satisfy host cell entry as well as the exchange of nutrition and metabolic waste. In this case, ALN-conjoined tetra-PEG hydrogel could enable the sustained release of ALN drugs, stem cell infiltration and substance exchange intra-extra of the hydrogel scaffolds. The compressive stress and modulus were important parameters to assess the mechanical stability of the hydrogels. As shown in Figure 3B, these tetra-PEG and ALN-conjoined tetra-PEG hydrogels exhibited the similar compressive behaviors with the analogous strength and modulus, further revealing the introduction of ALN didn’t affect the uniform network and the mechanical strength of tetra-PEG hydrogels. Besides, rheological experiment was applied to investigate the formation of ALN-conjoined tetra-PEG hydrogel and confirmed its high mechanical property (Figure 3C), which indicated its great potential for the regeneration of nonload-bearing bone defects such as cranial or nonunion defects.

### *In vitro* drug release

**FIGURE 4:**
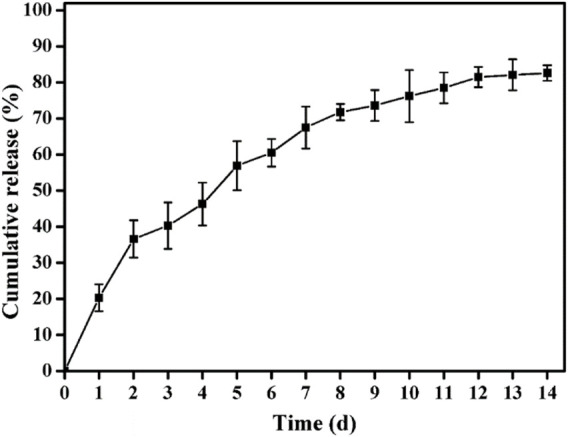
The release behavior from ALN-conjoined tetra-PEG hydrogel.

The release profile of ALN laden in the ALN-conjoined tetra-PEG hydrogel *in vitro* was assessed in Figure 4. A sustained release of ALN drug was observed for more than 2 weeks, which was due to the synergistic effect of physical encapsulation and chemical linkage of ALN drugs to control the sustainable drug release. In the first 2 days, a cumulative burst release of ALN drug quickly reached round 36%, which should be ascribed to the escaped free hydrophilic ALN drug from the hydrogel. Then the release contents approached its plateaus with nearly 80% at day 12, indicating the slow hydrolysis effects and the correspondingly sustained original ALN release into the solutions. This programmable and gradient release behavior not only satisfy the initial drug concentration to activate the bone repair mechanism in the defect areas but also conduct a sustained drug release to exert the long-term effects on sufficiently and persistently promoting the bone repair and tissue regenerations.

### Cell viability and proliferation

**FIGURE 5:**
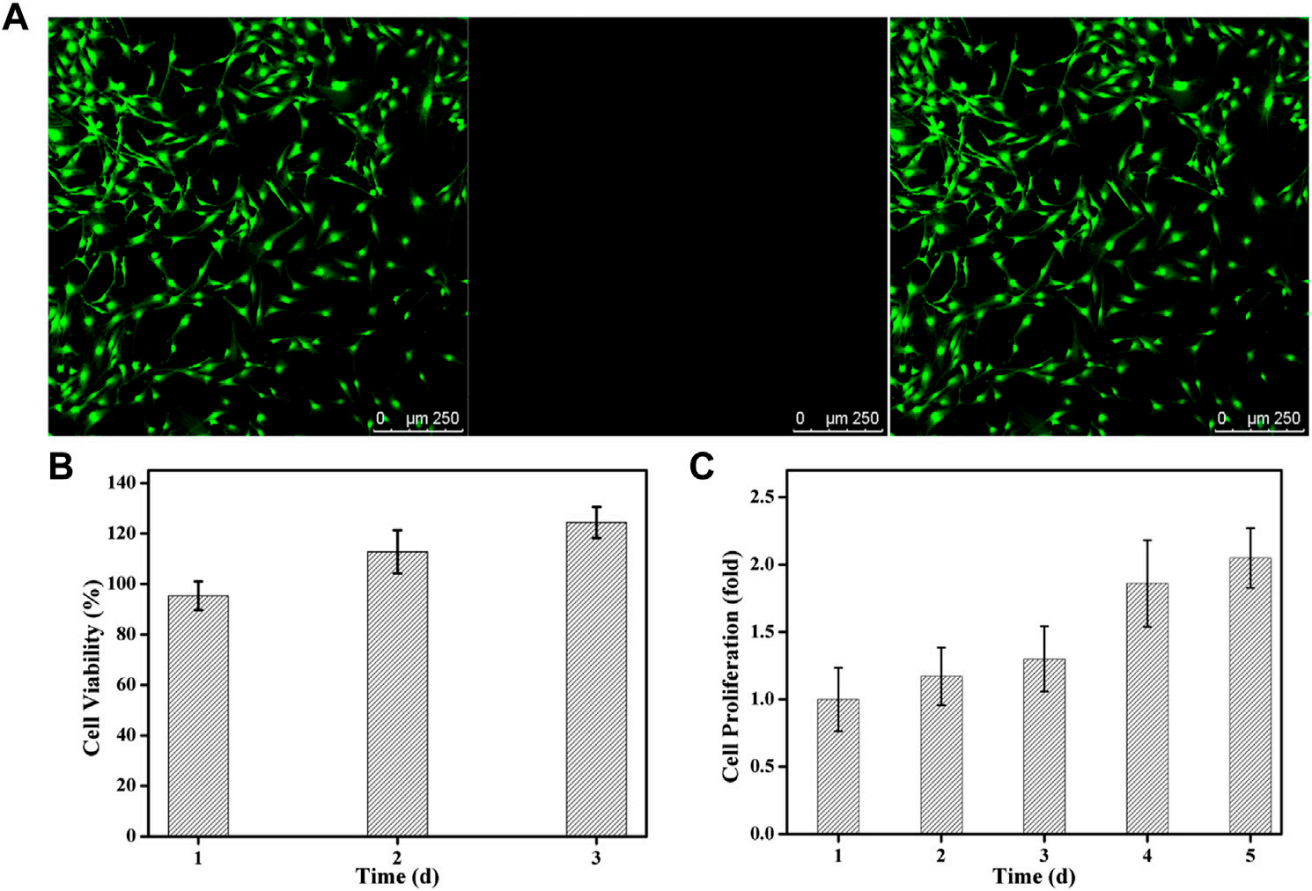
Cytotoxicity of ALN-conjoined tetra-PEG hydrogel *in vitro*. **(A)** Live/dead staining of PDLSCs. Cells in green manifest living PDLSCs while cells in red manifest dead ones. **(B)** Cell viability and **(C)** Cell proliferation was detected after cultivation for various time.

To the best of our knowledge, the FDA-approved PEG is nontoxic and used extensively in engineering applications and regenerative medicine like articular cartilage, neural, bladder and bone tissue repair. Therefore, cytotoxicity assay was carried out to testify the biosafety of ALN-conjoined tetra-PEG hydrogel using the PDLSCs. Live/dead assay confirmed that hydrogel scaffold possessed good biocompatibility corresponding to the high viability of PDLSCs after *in vitro* culture for 24 h (Figure 5A). Quantitatively, this ALN-conjoined tetra-PEG hydrogel was able to maintain high cell survival rate and promote the cell growth after 3 days of culture (Figure 5B), demonstrating the low cytotoxicity on PDLSCs that can be regarded as a preliminary indication of implanted scaffolds in the multiple biomedical and healthcare applications.

To investigate if this hydrogel was able to support the cell growth and proliferation, a long-term proliferation of CCK8 assay was conducted and OD value at 450 nm was measured at 1, 2, 3, 4 and 5 days after culture with ALN-conjoined tetra-PEG hydrogel. Since the cell number was correlated with optical density, the measurement of OD value at 450 nm could indicate the cell proliferation and growth. Figure 5C further testified the excellent biocompatibility of hydrogel scaffolds at these time points without significant difference. Cell proliferation rate was slightly increased in the initial 3 days after co-culturing with ALN-conjoined tetra-PEG hydrogel and showed a significant increase at day 4 and day 5 of incubation, revealing the favorable cell viability, growth and proliferation capacity of this kind of ALN-conjoined tetra-PEG hydrogel.

### Osteogenic differentiation of PDLSCs in the ALN-conjoined tetra-PEG hydrogel *in vitro*

**FIGURE 6:**
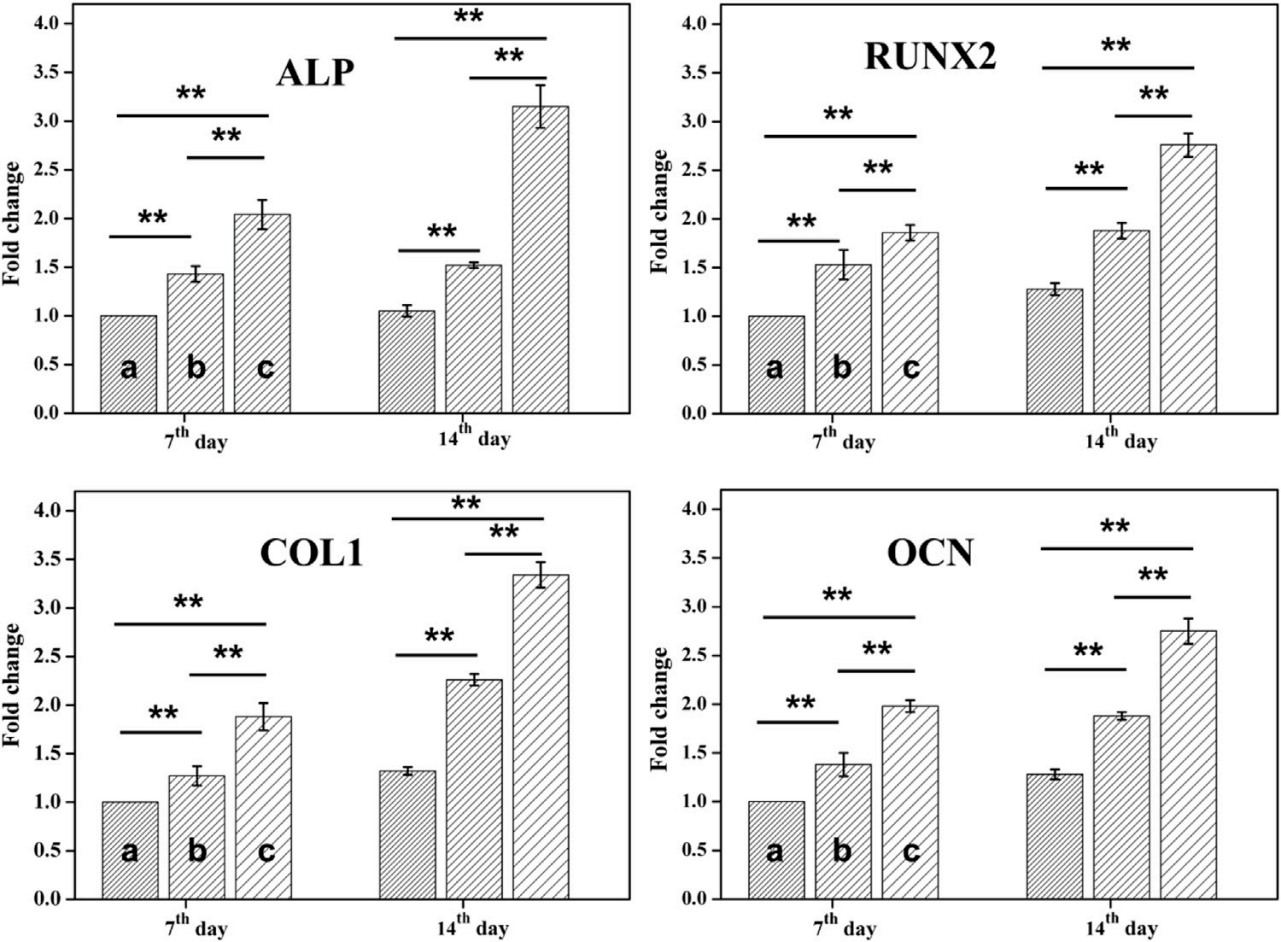
RT-PCR analysis. mRNA analysis of osteogenic markers of ALP, Runx2, COL1 and OCN by RT-PCR tests on day 7 and 14 (*n* = 3, *p < 0.05, **p < 0.01). Statistically significant differences in comparison with **(A)** control (untreated cells), **(B)** tetra-PEG hydrogel and **(C)** ALN-conjoined tetra-PEG hydrogel.

Ideal engineering bone repair scaffolds should enhance the osteogenic differentiation. To reveal the effect of hydrogel scaffolds on the osteogenic differentiation *in vitro*, we seeded the PDLSCs cells on the control, tetra-PEG hydrogel and ALN-conjoined tetra-PEG hydrogel. PCR assay was used to examine their osteoinduction abilities. As shown in Figure 6, Real-time PCR showed that the expression of osteogenesis-related genes in the tetra-PEG and ALN-conjoined tetra-PEG hydrogels were higher than that in the control group. The mRNA levels of osteogenic markers of typical ALP, Runx2, COL1 and OCN on the cell inoculated in the ALN-conjoined tetra-PEG hydrogel was higher than that in the tetra-PEG group, suggesting that the ALN drugs could effectively promote osteogenic differentiation of PDLSCs for a long period *in vitro*. In addition, this long-term osteogenic effect also revealed the importance of sustainable ALN release through a two-stage strategy. In other words, the encapsulated ALN released rapidly at an early period to reach high drug concentration to exert its osteogenesis, and the conjugated ALN provided a longer release period and prolonged the treatment time, resulting in enhanced therapeutic ossification effect, which was consistent with the previous works about bisphosphonates that can promote the expression levels of osteogenic-related genes in osteoblasts and improve osteogenic differentiation of stem cells.

## Conclusion

In summary, we developed an alendronate-conjoined tetra-PEG hydrogel with a sustained drug release behavior by simply mixing precursor together in solutions. This ALN-conjoined tetra-PEG hydrogel exhibited the advantageous traits such as homogeneously architectural networks, porous structures, potentially injectable behaviors, excitingly high mechanics and excellent cell proliferation capacity. On account of the simultaneous physical encapsulation and chemical linkage of the ALN within the hydrogels, this smart system allowed the rapid free ALN release at an early period to reach high drug concentration to exert its osteogenesis and longer release period of conjugated ALN, thus performing a programmable sustained release behavior and elucidating the therapeutic efficiency on preferentially guiding them toward osteogenic differentiation *in vitro*. Consequently, we believe that this current work is of great benefit to not only broaden the research minds for acquiring the perception to illustrate structure-property relationships, but also provide a generalized approach to achieving drug release regulation, therapy innovation and tissue regeneration enhancement.

## Data Availability

The raw data supporting the conclusions of this article will be made available by the authors, without undue reservation.
